# Analysis of proteins released from osteoarthritic cartilage by compressive loading

**DOI:** 10.1038/s41598-023-45472-x

**Published:** 2023-10-25

**Authors:** Hirotaka Tsuno, Nobuho Tanaka, Masashi Naito, Satoru Ohashi, Mitsuyasu Iwasawa, Tomoyasu Kadoguchi, Hiroyuki Mitomi, Toshihiro Matsui, Hiroshi Furukawa, Naoshi Fukui

**Affiliations:** 1 Clinical Research Center, National Hospital Organization Sagamihara Hospital, Sagamihara, Kanagawa Japan; 2 Department of Orthopaedic Surgery, National Hospital Organization Sagamihara Hospital, Sagamihara, Kanagawa Japan; 3 Department of Life Sciences, Graduate School of Arts and Sciences, University of Tokyo, Komaba 3-8-1, Meguro-ku, Tokyo, 153-8902 Japan; 4 Department of Diagnostic Pathology, Odawara Municipal Hospital, Odawara, Kanagawa Japan; 5 Clinical Research Center, National Hospital Organization Tokyo Hospital, Kiyose, Tokyo, Japan

**Keywords:** Musculoskeletal system, Rheumatic diseases

## Abstract

In osteoarthritis (OA), synovial pathology may be induced by proteins released from degenerated cartilage. This study was conducted to identify the proteins released from OA cartilage. OA cartilage was obtained from OA knees at macroscopically preserved areas (PRES) and degenerated areas (DEG), while control cartilage (CONT) was collected from non-arthritic knees. Released proteins were obtained from these cartilage samples by repeatedly applying compressive loading, which simulated loading on cartilage in vivo. The released proteins were analyzed comprehensively by antibody array analyses and a quantitative proteomic analysis. For several proteins, the exact amounts released were determined by Luminex assays. The amount of active TGF-β that was released was determined by an assay using genetically-engineered HEK cells. The results of the antibody array and proteomic analyses revealed that various biologically active proteins are released from OA cartilage, particularly from DEG, by loading. The Luminex assay confirmed that several alarmins, complement proteins C3a and C5a, and several angiogenic proteins including FGF-1, FGF-2 and VEGF-A were released in greater amounts from DEG than from CONT. The HEK cell assay indicated that active TGF-β was released from DEG at biologically significant levels. These findings may be helpful in understanding the pathology of OA.

## Introduction

In osteoarthritis (OA), synovium undergoes obvious changes. Although once viewed as a secondary change associated with cartilage degeneration, synovial changes are now considered to play a key role in the pathology of OA. The results of epidemiological studies have shown that the synovial changes are closely associated with the progression and symptoms of knee OA^1, 2^. However, despite its significance, the mechanism(s) underlying the changes remains to be determined.

At present, it is widely accepted that synovial changes are induced by factors released from degenerated cartilage^1, 3–5^. This view is supported by the results of arthroplasty surgery. Synovial changes in knee OA usually resolve after the removal of the entire cartilage by total knee arthroplasty. Considering the favorable results of unicompartmental knee arthroplasty, removal of degenerated cartilage may be sufficient for resolution. As these observations imply the involvement of proteins released from degenerated cartilage in the perpetuation of synovial pathology, several investigators have analyzed such proteins under various conditions^6^. However, few studies have the analyzed proteins directly released from OA cartilage tissues under physiological levels of loading.

In this study, we performed a comprehensive analysis of the proteins released from degenerated cartilage. In such analyses, it may be an issue how released proteins are obtained from cartilage tissues. In weight-bearing joints, cartilage is subjected to loading multiple times daily. Because articular cartilage is composed of a porous matrix, interstitial fluid within the matrix may be compressed out by the loading^7^. Then, it is plausible that the proteins within the cartilage may be released by loading, together with the fluid. In this study, we applied a physiological level of compressive loading to cartilage tissues, and analyzed the proteins released from these tissues. Analyses revealed that various biologically active proteins are released from OA cartilage by loading close to that given during daily activities.

## Materials and methods

### Acquisition of cartilage and synovial tissues

**Figure 1:**
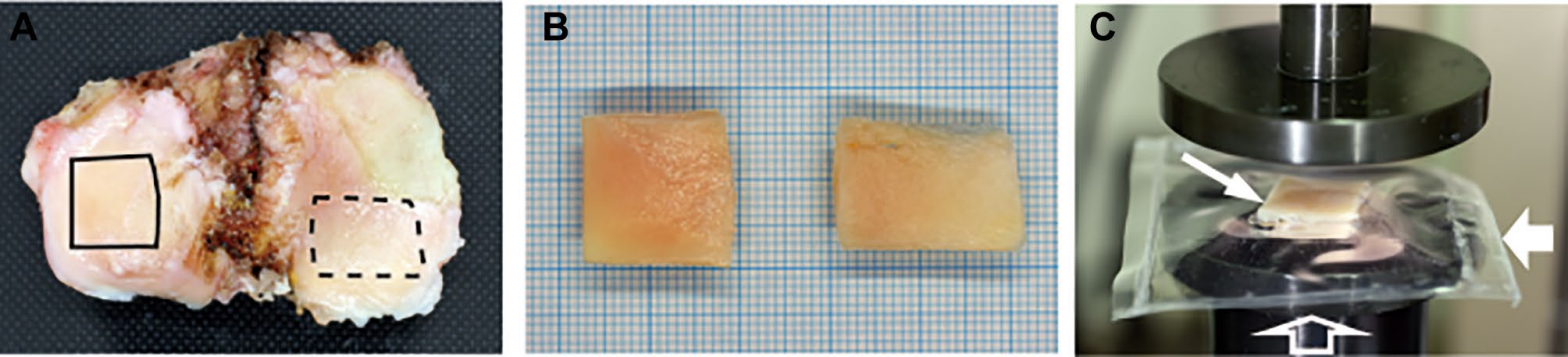
Obtaining OA cartilage and loading of cartilage tissues. (**A**) Tibial condyles were obtained from a medially involved OA knee at surgery, and PRES and DEG were obtained from a macroscopically preserved area on a lateral condyle (solid line) and a degenerated area on a medial condyle (broken line), respectively. (**B**) Acquired cartilage was placed on a graph paper and photographed to determine a surface area. (**C**) Cartilage tissue (thin arrow) was placed in a plastic bag (bold arrow) with a corresponding amount of PBS, which was heat-sealed and placed on a platform of a universal testing machine (open arrow) for loading.

This study was conducted in accordance with the Declaration of Helsinki and was approved by the Institutional Review Committees of the National Hospital Organization Sagamihara Hospital and the Odawara Municipal Hospital, and no samples were collected from prisoners. Prior to acquisition of cartilage samples, written informed consent was obtained from each patient or the family of the donor. OA cartilage samples were obtained from 62 end-stage OA knee joints of 62 patients (9 males and 53 females; mean age, 75.8 [range 58–88] years) at prosthetic surgery. The diagnosis of OA was based on the established criteria for knee OA^8^. All knees were medially involved in the disease, and the knees with lateral or bilateral involvement were not included in this study. In each OA knee, cartilage samples were obtained in a pair from a macroscopically preserved area (PRES) and a degenerated area (DEG) on the tibial plateau (Fig. 1A). At those areas, cartilage tissues were cut immediately above the subchondral bone using a scalpel. Contamination of the bone or other fibrous tissues was carefully avoided, which was later confirmed by histology. In 3 knees, synovial tissues were also obtained for an experiment using primary cultured synovial cells.

Control cartilage samples (CONT) were obtained at dissection from 9 non-arthritic knee joints of 9 donors (4 males and 5 females; mean age, 80.1 [range, 63–95] years) within 24 h after death. The donors had no known history of joint diseases or serious trauma, and the cartilage samples were confirmed to show no apparent signs of degeneration. In those knees, cartilage samples were obtained at 2–4 sites on the tibial plateaus in areas corresponding to those of the OA cartilage.

After they were obtained, the cartilage samples were rinsed extensively with ice-cold PBS at least 3 times. The samples were then blotted, weighed, photographed on a graph paper (Fig. 1B), and stored at  − 80 °C in a plastic film until use. The photograph was incorporated into a computer, and the tissue surface area was determined using the ImageJ software (version 1.53; NIH, Bethesda, MD, USA). Cartilage samples were carefully handled to avoid possible contamination throughout the experiments.

### Obtainment of released proteins from cartilage tissues

In order to obtain proteins released from cartilage by loading, cartilage tissue was placed in a heat-sealable plastic bag together with the corresponding amount of phosphate-buffered saline (PBS; 2.5 ml/g wet weight of cartilage). A proteinase inhibitor cocktail (Promega, Madison, WI, USA) was added to the PBS at the concentration indicated by the manufacturer. For cell culture experiments, culture media were used instead of PBS without the addition of the proteinase inhibitor cocktail.

The bag was then heat-sealed and placed flat on the platform of a universal testing machine (Model SV-201NA, Imada Seisakusyo, Toyohashi, Aichi, Japan) equipped with a load cell (Model LC-050N, Imada Seisakusyo) and a stainless-steel crosshead (55 mm in diameter) with a flat surface (Fig. 1C).

A load of 1 MPa was applied to the tissue 60 times repeatedly, simulating the load on the cartilage in vivo during level walking^9^. To perform this loading, the crosshead was programmed to descend at a rate of 50 mm/min until a load of 1 MPa (10.2 kg/cm^2^) was applied to the tissue. Upon reaching this load, the crosshead was set to elevate upwards immediately, at the same speed, until the tissue was completely unloaded. This loading cycle was repeated 60 times consecutively, which required 2–9 min depending on the elasticity and thickness of the tissue. After loading, the PBS in the plastic bag was recovered, clarified by centrifugation, filtered through a 0.22-µm-pore polyethersulfone filter (Millipore Express; Millipore, Burlington, MA, USA), and stored in aliquots at  − 80 °C until use. The cartilage tissues were recovered and processed for histological evaluation. For comparison, some tissues were left to stand without loading for 10 min, and then the PBS and cartilage were recovered and processed in the same manner.

### Histological evaluation

After loading, cartilage tissues from OA knees (PRES and DEG) were examined histologically. The details are described in the Supplementary Methods. Evaluation was performed according to the Osteoarthritis Research Society International (OARSI) histological grades^10^.

### Comparison of the amounts and profiles of released proteins

The amounts of proteins released from the cartilage tissues were determined using a Pierce BCA Protein Assay Kit (ThermoFischer Scientific, Waltham, MA, USA). The profiles of the proteins released by loading were compared among CONT, PRES and DEG using sodium dodecyl sulfate–polyacrylamide gel electrophoresis (SDS-PAGE). For the analysis, 1 μg of released proteins was loaded on a 5–20% SDS-PAGE gel (XV Pantera, DRC, Tokyo, Japan), and proteins were resolved by electrophoresis. The gel was silver-stained to visualize protein bands using a commercially available kit (2D-Silver Stain Reagent II, Cosmo Bio, Tokyo, Japan), in accordance with the manufacturer’s instructions.

### Antibody array analysis

Proteins released from cartilage tissues were analyzed using 4 commercially available antibody arrays (Proteome Profiler Human Adipokine, Human Angiogenesis, Human Chemokine, and Human XL Cytokine Array Kits; R&D Systems, Minneapolis, MN, USA), in accordance with the manufacturer’s instructions. Briefly, after blocking, the array was incubated with 0.5 ml of PBS containing released proteins overnight at 4 °C on a rocking platform shaker. The array was washed 3 times with washing buffer to remove unbound proteins, and incubated with a cocktail of biotinylated detection antibodies for 2 h at room temperature. Streptavidin-HRP and chemiluminescent detection reagents were then applied, and a signal was generated at each antibody spot on the array membrane with an intensity corresponding to the amount of bound protein. The signals were detected by a Typhoon 9410 imager (GE Healthcare, Chicago, IL, USA) and quantified by ImageJ (version 1.53r, National Institutes of Health, Bethesda, MD, USA).

### Quantitative proteomic analysis using iTRAQ labelling

Proteins released from cartilage tissues by loading were subjected to LC-MS/MS analysis with isobaric tags for relative and absolute quantification (iTRAQ) labelling following a previously described method with some modifications^11^. Two CONT and two DEG samples obtained from two donors and two patients, respectively, were used for this analysis. The details of this analysis are described in the Supplementary Methods.

### Functional enrichment analysis

The functional implication of the proteins released from DEG in greater amounts in comparison to CONT was explored using Gene Ontology (GO) and Kyoto Encyclopaedia of Genes and Genomes (KEGG) pathway enrichment analyses with the assistance of DAVID Bioinformatics Resources (v6.8, https://david.ncifcrf.gov/). *P* < 0.01 was used as the threshold for both analyses.

### Luminex assay

For some proteins, the amounts released from cartilage tissues were quantified by bead-based suspension array technology on a Luminex 100/200 analyzer (Luminex, Austin, TX, USA) using commercially available kits (Magnetic Luminex Performance Assay and Human Luminex Discovery Assay, R&D Systems, and C3a Human ProcartaPlex Simplex kit, ThermoFisher Scientific), according to the manufacturers’ instructions. Briefly, 50 µl of PBS containing the released proteins was incubated with a suspension of capture antibody-conjugated magnetic microspheres. The microspheres were then incubated with a biotinylated detection antibody and streptavidin–phycoerythrin conjugate, and fluorescence intensity was measured. For the measurement of TGF-β1, β2 and β3, samples were acid-activated prior to measurement using Sample Activation Kit 1 (R&D Systems).

### Determination of TGF-β activity

The amount of biologically active TGF-β released from cartilage tissues by loading was determined by an assay using HEK-Blue TGF-β cells (InvivoGen, San Diego, CA, USA). The details are described in the Supplementary Methods. Briefly, the released proteins were obtained from 8 pairs of PRES and DEG from 8 OA knees and 6 CONT from 5 control knees. HEK-Blue cells were cultured in media containing either released proteins or graded concentrations of recombinant human TGF-β1 (rhTGF-β1, R&D Systems) for 24 h. Then the media were recovered, and the amounts of active TGF-β in the media were determined based on the activities of secreted embryonic alkaline phosphatase (SEAP) released from the HEK-Blue cells, using rhTGF-β1 as a standard.

### Experiment using primary cultured synovial cells

This experiment is described in detail in the Supplementary Methods. Briefly, primary cultured synovial cells were obtained from synovial tissues collected from 3 end-stage OA knees by enzymic digestion, following a previously described method^12^ with some modifications. Following digestion, cells were cultured in media containing released proteins with or without SB431542 (Sigma-Aldrich, St. Louis, MO, USA), a specific inhibitor of TGF-β type 1 receptor. Twenty-four hours later, RNA was obtained, cDNA was generated, and the expression levels of urokinase and plasminogen activator inhibitor-1 (PAI-1) were determined by qPCR using β-actin as an internal standard.

### Statistical analysis

Statistical differences were determined by the Kruskal–Wallis test followed by Scheffe’s post hoc test. Unless otherwise indicated, *p* values of < 0.05 were considered to indicate statistical significance.

## Results

### Substantial amounts of proteins were released from cartilage tissues by compressive loading

**Figure 2:**
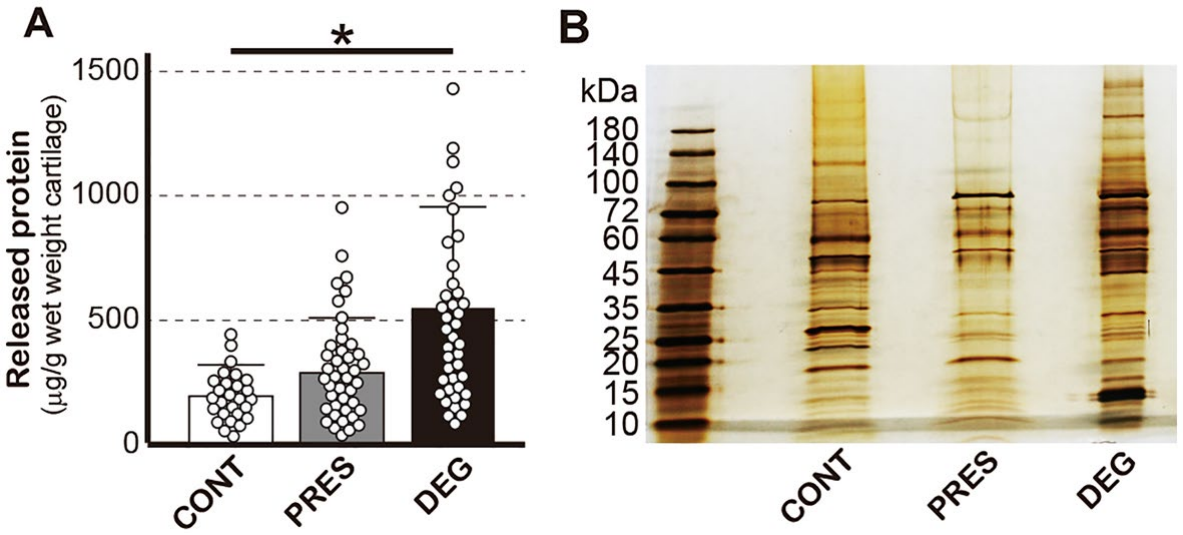
(**A**) Amounts of proteins released from cartilage tissues by compressive loading. Bars show the mean + SD. (**B**) Result of SDS-PAGE of the released proteins. Proteins were visualized by silver-staining. The analysis was repeated four times using respective samples, and a representative result is shown. In A and B, CONT, PRES and DEG denote control cartilage, and OA cartilage from preserved areas and degenerated areas, respectively.

A total of 33 CONT and 54 pairs of PRES and DEG were used to obtain proteins released by loading. The surface area and wet weight of these cartilage tissues did not differ significantly among CONT, PRES and DEG, whereas the OARSI scores of DEG were significantly greater than those of PRES (Table 1 and Supplementary Fig. S1). We first determined the amounts of proteins released from cartilage tissues by compressive loading. The amounts of proteins released from CONT were the lowest (181 ± 125 μg/g), followed by PRES (273 ± 218 μg/g), while the greatest amounts were released from DEG (531 ± 400 μg/g), and the amount of proteins released from DEG was significantly greater than that from CONT (Fig. 2A). Because the amounts of released proteins were < 10% when loading was not applied to the tissues (Supplementary Table S1), the major parts of those proteins were considered to have been released by compressive loading.

**Table 1:** Cartilage tissues used for the study.

Cartilage	Surface area (cm^2^)	Wet weight (mg)	OARSI score
CONT	2.66 ± 0.75	488 ± 174	N/A
PRES	2.75 ± 0.55	527 ± 273	1.43 ± 0.50
DEG	2.57 ± 0.93	448 ± 173	2.70 ± 0.63***

CONT, PRES and DEG denote control cartilage, and OA cartilage obtained from macroscopically presereved areas and degenerated areas, respectively. Results represent the mean ± SD of 33 (CONT) or 54 cartilage tissues (PRES and DEG). ***P < 0.001 against PRES by Wilcoxon signed-rank test.

We then performed SDS-PAGE and compared the profiles of released proteins among the three cartilage groups. This analysis revealed differences in band patterns among the three groups (Fig. 2B), which prompted us to perform comparisons of released proteins among these groups.

### Osteoarthritic cartilage releases various biologically active proteins by compressive loading

The proteins released from cartilage tissues were first subjected to antibody array analysis. Eighteen cartilage tissues from each cartilage group were used for this analysis (Supplementary Table S2). In this analysis, we focused on the proteins released from DEG in greater amounts in comparison to PRES and CONT, since such proteins are more likely to be involved in synovial pathology.

**Figure 3:**
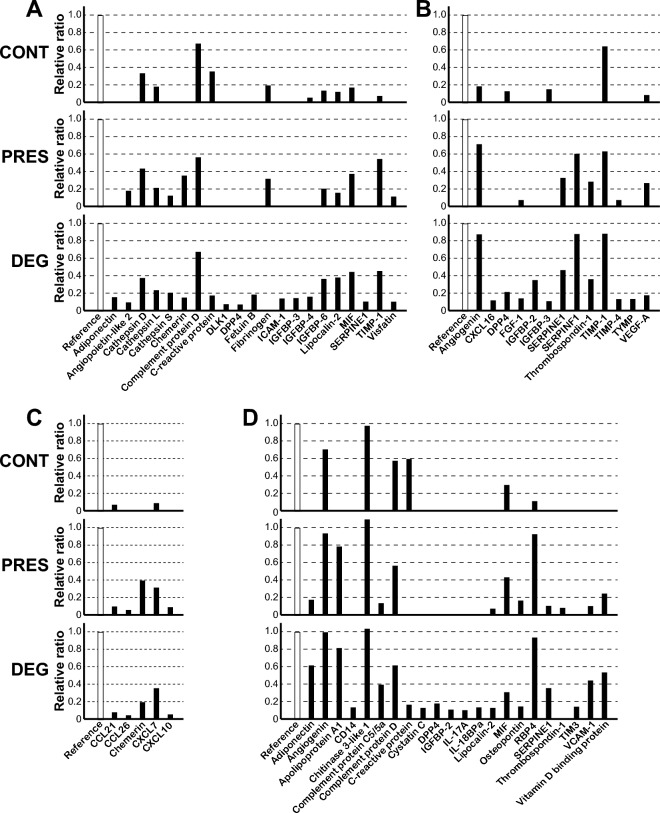
Results of the antibody array analysis. Cartilage tissues obtained from control knees (CONT) and preserved (PRES) or degenerated areas (DEG) of OA knees were subjected to repetition of compressive loading, and released proteins were analyzed using four types of antibody arrays. The results of densitometric measurements of the arrays focused on adipokines and related proteins (**A**), angiogenesis-related proteins (**B**), chemokines (**C**) and cytokines and related proteins (**D**) are shown. For each type of array, the analysis was repeated 4 times (Adipokine, Chemokine and XL Cytokine arrays) or 6 times (Angiogenesis array), and representative results are shown. The analyses were performed using respective cartilage tissues, and no samples were used for more than one membrane. In each graph, bars represent relative ratios of the signal intensities of antibody spots against that of the reference spot on that membrane.

The analysis using the array focused on adipokines and related proteins revealed that adiponectin, DLK1, DPP4, fetuin-B, ICAM-1, IGFBP-3 and SERPINE1 were detected only with proteins released from DEG under our experimental conditions (Fig. 3A and Supplementary Fig. S2A). Besides these proteins, IGFBP-6, lipocalin-2 and MIF were released in greater amounts from DEG than from CONT and PRES.

The array targeting angiogenesis-related proteins revealed that CXCL16, IGFBP-2 and TYMP were released only from DEG, and three other proteins, DPP4, FGF-1 and TIMP-4 were released in greater amounts from DEG than from CONT and PRES (Fig. 3B and Supplementary Fig. S2B). Besides these, angiogenin, SERPINE1, SERPINF1, thrombospondin-1 and VEGF-A were released from PRES and DEG in similar amounts, whereas limited amounts were released from CONT.

The analysis using the array focused on chemokines revealed an obvious difference in the released proteins between OA and control cartilage. While five chemokines were found to be released from PRES and DEG, only two of them were released from CONT in smaller amounts. The profiles of released proteins were very similar between PRES and DEG, and both types of cartilage released chemerin and CXCL7 rather abundantly (Fig. 3C and Supplementary Fig. S2C).

Finally, in the analysis using the array for cytokines and related proteins, a total of 21 proteins were identified to be released from DEG by loading (Fig. 3D and Supplementary Fig. S2D). Among them, 13, including adiponectin, complement protein C5/5a, IL-17A and SERPINE1, were released in greater amounts from DEG than from PRES and CONT. Three other proteins, apolipoprotein A1, osteopontin and RBP4 were released in similar amounts from PRES and DEG whereas they were little released from CONT.

Among the four antibody arrays used above, the antibody spots for 11 proteins were contained in two or three arrays. The comparison of the spot densities for these proteins across the arrays indicated reasonable reproducibility of the analysis (Supplementary Fig. S3).

In order to understand the significance of the antibody array analysis, we chose proteins for which the spot density for DEG was ≥ 2 times that of CONT. These proteins were further divided by the difference in spot densities between PRES and DEG, that is, proteins for which the density of DEG was ≥ 2 times that of PRES (Table 2), and others (Table 3), assuming that the former are more likely to be involved in OA pathology.

**Table 2:** Proteins for which the spot densities of DEG were two times or greater those of CONT and PRES.

Antibody array	Adipokines and related proteins	Angiogenesis-related proteins	Chemokines	Cytokines and related proteins
Identified proteins	Adiponectin	CXCL16		Adiponectin
	DLK1	FGF-1		CD14
	DPP4	IGFBP-2^d^		Complement protein 5/5a
	Fetuin B	TIMP-4		Cystatin C
	ICAM-1	TYMP		DPP4
	IGFBP-3^a^			IGFBP-2
	IGFBP-4			IL-17A
	Lipocalin-2^b^			IL-18BPa
	SERPINE1^c^			Lipocalin-2^b^
				SERPINE1^c^
				TIMP3
				VCAM1
				Vitamin D binding protein

^a^Not identified by the array for angiogenesis-related proteins.

^b^Identified equally by the array for adipokines and related proteins and that for cytokines and related proteins.

^c^Identified by the array for adipokines and related proteins and that for cytokines and related proteins, but not identified by the array for angiogenesis-related proteins.

^d^Not identified by the array for adipokines and related proteins.

**Table 3:** Proteins for which the spot densities of DEG were two times or greater those of CONT but not two times those of PRES.

Antibody array	Adipokines and related proteins	Angiogenesis-related proteins	Chemokines	Cytokines and related proteins
Identified proteins	Angiopietin-like 2	Angiogenin^a^	CCL26	Apolipoprotein A1
	Cathepsin S	SERPINE1^c^	Chemerin	Osteopontin
	Chemerin	SERPINF1	CXCL7	RBP4
	MIF^a^	Thrombospondin-1^d^	CXCL10	
	TIMP-1^b^	VEGF-A		
	Visfatin			

^a^Not identified by the array for cytokines and related proteins.

^b^Not identified by the array for angiogenesis-related proteins.

^c^Not identified by the array for adipokines and related proteins or that for cytokines and related proteins.

^d^Not identified by the array for cytokines and related proteins.

### The quantitative proteomic analysis revealed the release of matrix components and alarmins from diseased cartilage by loading

Since the antibody array analysis revealed that various proteins were released from cartilage tissues by loading, we wished to determine the released proteins more comprehensively. We therefore performed a quantitative proteomic analysis using iTRAQ labelling. Proteins released from two CONT and two DEG samples were subjected to this analysis, and a total of 535 proteins were identified at a 1% false discovery rate (FDR). All proteins identified in this analysis are listed in Supplementary Table S3.

Based on these results, we again chose proteins that were released from DEG in greater amounts in comparison to CONT on the condition that the amounts released from the two DEG samples were both greater in comparison to the two CONT samples, and that the mean of the amounts released from the two DEG samples was ≥ 2 times that of the two CONT samples.

Among the 535 proteins identified, 58 fulfilled these criteria. The top 20 proteins, according to the DEG to CONT ratio, are shown in Table 4, with the rest shown in Supplementary Table S4. As anticipated, approximately half of the top 20 proteins were components of the cartilage matrix and related molecules, likely reflecting their degeneration and/or enhanced synthesis in the diseased cartilage. Again, it should be noted that among those listed, cartilage oligomeric matrix protein (COMP), fibronectin, lumican, fibromodulin, biglycan and decorin are known members of alarmins or damage-associated molecular patterns (DAMPs)^4, 13–15^.

**Table 4:** Proteins identified to be released in greater abundance from DEG compared to PRES by a quantitative proteomic analysis.

Rank	Accession	Description	Coverage (%)	Peptides	Abundance				Relative ratio
					CONT1	CONT2	DEG1	DEG2	
No. 1	O43707	Alpha-actinin-4	2.4	2	195,900	330,600	2,080,000	2,967,000	9.586
No. 2	P49747	Cartilage oligomeric matrix protein	71.5	46	344,900,000	283,800,000	2.543E + 09	1.501E + 09	6.432
No. 3	P02751	Fibronectin	44.0	81	268,600,000	316,100,000	1.423E + 09	2.263E + 09	6.304
No. 4	P51884	Lumican	49.7	16	100,900,000	104,300,000	966,900,000	290,600,000	6.128
No. 5	P02679	Fibrinogen gamma chain	40.0	22	128,000,000	104,800,000	553,200,000	485,000,000	4.460
No. 6	Q06828	Fibromodulin	35.4	11	28,720,000	33,490,000	189,500,000	73,550,000	4.228
No. 7	Q92765	Secreted frizzled-related protein 3	4.0	1	9251	23,670	26,780	109,000	4.124
No. 8	P61626	Lysozyme C	72.3	12	45,410,000	75,130,000	318,800,000	176,200,000	4.107
No. 9	P02675	Fibrinogen beta chain	56.8	27	172,800,000	119,700,000	638,700,000	547,300,000	4.055
No.10	P02458	Collagen alpha-1(II) chain	18.0	26	46,540,000	57,630,000	345,200,000	67,440,000	3.961
No.11	Q99983	Osteomodulin	15.0	6	4,747,000	5,389,000	27,450,000	12,400,000	3.932
No.12	Q08345	Epithelial discoidin domain-containing receptor 1	1.3	1	306,900	942,700	3,428,000	1,288,000	3.774
No.13	P02452	Collagen alpha-1(I) chain	14.6	18	8,711,000	14,050,000	58,960,000	22,040,000	3.559
No.14	P21810	Biglycan	46.5	14	31,070,000	38,410,000	187,800,000	55,790,000	3.506
No.15	O15335	Chondroadherin	63.5	19	38,670,000	48,070,000	225,300,000	65,550,000	3.353
No.16	P02743	Serum amyloid P-component	35.9	9	18,130,000	27,550,000	64,140,000	88,800,000	3.348
No.17	P02671	Fibrinogen alpha chain	41.9	35	195,800,000	222,300,000	714,000,000	679,100,000	3.332
No.18	P07585	Decorin	46.5	18	152,700,000	326,600,000	1.177E + 09	407,500,000	3.306
No.19	Q15113	Procollagen C-endopeptidase enhancer 1	44.8	18	17,430,000	15,380,000	83,690,000	23,530,000	3.268
No.20	P69905	Hemoglobin subunit alpha	62.7	8	132,100,000	77,270,000	365,000,000	311,000,000	3.229

Accession is the UniProt accession number. Coverage is the ratio of the number of amino acids in all identified peptides relative to the number of amino acids in the entire protein. Peptides are the number of identified peptides for the protein. CONT1, CONT2, DEG1 and DEG2 denote abundances of respective cartilage samples. Relative ratio is the ratio of the sum of the abundances of two DEG samples relative to that of two CONT samples. Proteins are ranked by relative ratio.

### The enrichment analysis revealed the characteristics of released proteins

Next, the characteristics of the above-selected 58 proteins were investigated by GO and KEGG pathway enrichment analyses using the DAVID Bioinformatics Resources. The results of GO enrichment analysis are shown in Table 5. In the category of biological processes, the selected proteins were most involved in collagen fibril organization according to the *p* value, followed by extracellular matrix organization and skeletal system development. In the terms related to molecular function annotations, extracellular matrix structural constituent was the most enriched, followed by collagen binding, and heparin binding. In the cellular components category, extracellular space and extracellular region were the most enriched terms.

**Table 5:** Results of GO enrichment analysis.

Category	Term	Protein count	%	*P* value	Fold enrichment
GOTERM_BP_DIRECT	GO:0030199 ~ collagen fibril organization	10	16.9	1.40E−13	54.4
	GO:0030198 ~ extracellular matrix organization	12	20.3	2.10E−12	23.7
	GO:0001501 ~ skeletal system development	11	18.6	8.64E−12	26.4
	GO:0072378 ~ blood coagulation, fibrin clot formation	4	6.8	9.00E−07	186.5
	GO:0070527 ~ platelet aggregation	5	8.5	1.01E−05	36.3
	GO:0007160 ~ cell–matrix adhesion	6	10.2	1.75E−05	18.3
	GO:0007155 ~ cell adhesion	9	15.3	2.50E−04	5.3
	GO:0090277 ~ positive regulation of peptide hormone secretion	3	5.1	3.95E−04	97.9
	GO:0045087 ~ innate immune response	9	15.3	3.98E−04	4.9
	GO:0007601 ~ visual perception	6	10.2	4.31E−04	9.3
GOTERM_MF_DIRECT	GO:0005201 ~ extracellular matrix structural constituent	22	37.3	2.07E−31	53.6
	GO:0005518 ~ collagen binding	11	18.6	4.96E−15	54.3
	GO:0008201 ~ heparin binding	12	20.3	2.94E−12	22.9
	GO:0030021 ~ extracellular matrix structural constituent conferring compression resistance	7	11.9	3.69E−12	147.0
	GO:0050840 ~ extracellular matrix binding	6	10.2	2.84E−08	65.0
	GO:0030020 ~ extracellular matrix structural constituent conferring tensile strength	6	10.2	1.23E−07	49.2
	GO:0048407 ~ platelet-derived growth factor binding	4	6.8	3.84E−06	122.1
	GO:0002020 ~ protease binding	6	10.2	1.65E−05	18.5
	GO:0005178 ~ integrin binding	6	10.2	1.06E−04	12.5
	GO:0016504 ~ peptidase activator activity	3	5.1	4.54E−04	91.6
GOTERM_CC_DIRECT	GO:0005615 ~ extracellular space	44	74.6	4.09E−32	7.9
	GO:0005576 ~ extracellular region	40	67.8	3.96E−25	6.5
	GO:0031012 ~ extracellular matrix	20	33.9	2.67E−22	26.8
	GO:0070062 ~ extracellular exosome	29	49.2	8.94E−13	4.5
	GO:0005788 ~ endoplasmic reticulum lumen	12	20.3	8.48E−10	13.6
	GO:0005581 ~ collagen trimer	8	13.6	7.32E−09	30.2
	GO:0072562 ~ blood microparticle	9	15.3	7.41E−09	21.5
	GO:0005796 ~ Golgi lumen	8	13.6	1.98E−08	26.2
	GO:0043202 ~ lysosomal lumen	7	11.9	3.02E−07	25.3
	GO:0005577 ~ fibrinogen complex	4	6.8	1.20E−06	173.5

In each category, top 10 terms by *p* value are shown. Protein count is the number of proteins identified by the analysis in a set of proteins annotated to the term, and percentage is the ratio of the number of identified proteins relative to that of all proteins annotated to the term.

KEGG pathway analysis revealed that the above-selected proteins were significantly enriched in a total of 10 pathways (Table 6). Among these, ECM-receptor interaction was the most enriched pathway according to the *p* value, followed by focal adhesion, complement, and coagulation cascades.

**Table 6:** Results of KEGG pathway enrichment analysis.

Term	Protein count	%	*P* value	Fold enrichment
hsa04512:ECM-receptor interaction	8	13.6	1.72E−08	24.7
hsa04510:focal adhesion	9	15.3	3.26E−07	12.2
hsa04610:complement and coagulation cascades	6	10.2	1.07E−05	19.2
hsa04974:protein digestion and absorption	6	10.2	2.72E−05	15.8
hsa04611:platelet activation	6	10.2	6.64E−05	13.2
hsa05165:human papillomavirus infection	8	13.6	1.23E−04	6.6
hsa04151:PI3K-Akt signaling pathway	8	13.6	1.86E−04	6.1
hsa05146:amoebiasis	5	8.5	4.31E−04	13.3
hsa04933:AGE-RAGE signaling pathway in diabetic complications	4	6.8	5.19E−03	10.9
hsa05205:proteoglycans in cancer	5	8.5	5.62E−03	6.6

Top 10 pathways by *p* value are shown. For descriptions, refer to Table 3.

### The quantitative analysis confirmed that various proteins are released in greater amounts from OA cartilage by loading

Since the above analyses indicated that, under compressive loading, various proteins were released in greater amounts from OA cartilage than from CONT, we next determined the exact amounts of release for several proteins of interest.

Among the proteins that had been found to be released in greater amounts from DEG, we chose adiponectin, complement protein C5a, FGF-1, fibronectin, IL-17A, MIF, VEGF-A and visfatin for quantification because their possible involvement in OA pathology has been previously reported^16–23^. In addition, we determined the amounts of protein released for 6 other proteins that had not been identified by the above analyses but could play certain roles in OA pathology. Among them, FGF-2 and TGF-β1, β2 and β3 were quantified because they are known to be retained within the cartilage matrix^24–26^, and could therefore be released upon cartilage degeneration. Furthermore, the amount of complement protein C3a released was determined based on its relationship with C5a. We also chose nerve growth factor (NGF) for quantification because it is a key molecule in OA pain^27^ and can be expressed by chondrocytes in OA cartilage^28^.

**Figure 4:**
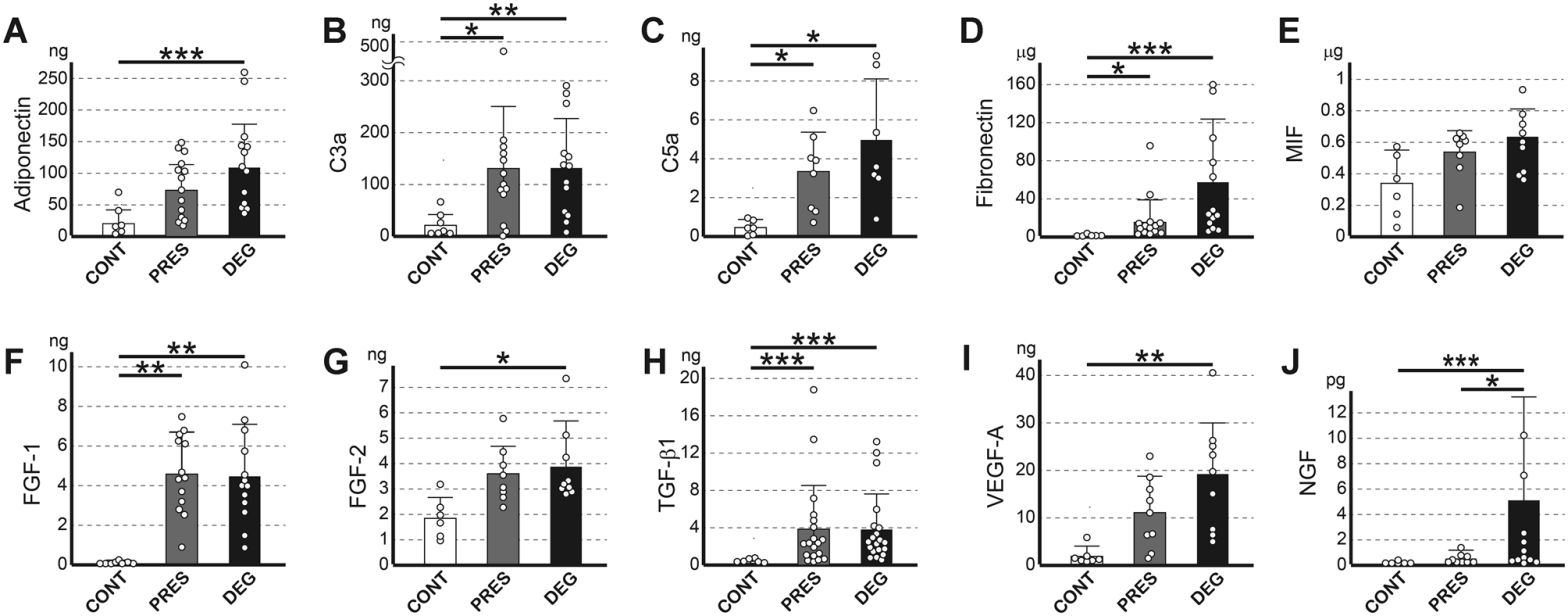
Results of the quantification of released proteins. The amounts of proteins released from control cartilage (CONT) and OA cartilage from preserved areas (PRES) or degenerated areas (DEG) were determined by an analysis using a Luminex analyzer. The results of adiponectin (**A**), complement protein C3a (**B**), C5a (**C**), fibronectin (**D**), MIF (**E**), FGF-1 (**F**), FGF-2 (**G**), TGF-β1 (**H**), VEGF-A (**I**) and NGF (**J**) are shown. Amounts are normalized by the wet weights of cartilage tissues. Open, shaded and solid bars are the results of CONT, PRES and DEG, respectively. Each bar represents mean and SD of 6–20 samples. *, ** and *** denote *p* < 0.05, 0.01 and 0.001, respectively.

Among the 14 above-mentioned proteins, IL-17A, visfatin, TGF-β2 and β3 were released from OA cartilage (PRES and DEG) at mostly undetectable levels (data not shown), and cartilage was not considered to be the source of those proteins in OA joints. Meanwhile, for another 9 proteins, the amounts released from DEG were significantly greater than those from CONT (Fig. 4). For C3a, C5a, fibronectin, FGF-1 and TGF-β1, the amounts released from PRES were also significantly greater than those from CONT. The amount of MIF released also tended to be greater in the order of CONT, PRES and DEG, although the differences among the groups were not statistically significant.

### Biologically active TGF-β is released in substantial amounts from OA cartilage by loading

In vivo, TGF-β is synthesized and secreted from cells in biologically inactive latent forms, and exhibits biological activities only after it is processed for activation^29^. Since the Luminex assay revealed that TGF-β1 was released from OA cartilage at substantial levels, we wished to determine whether the released protein was biologically active. To this end, we conducted a biological assay using HEK cells genetically engineered to secrete SEAP in response to TGF-β.

**Figure 5:**
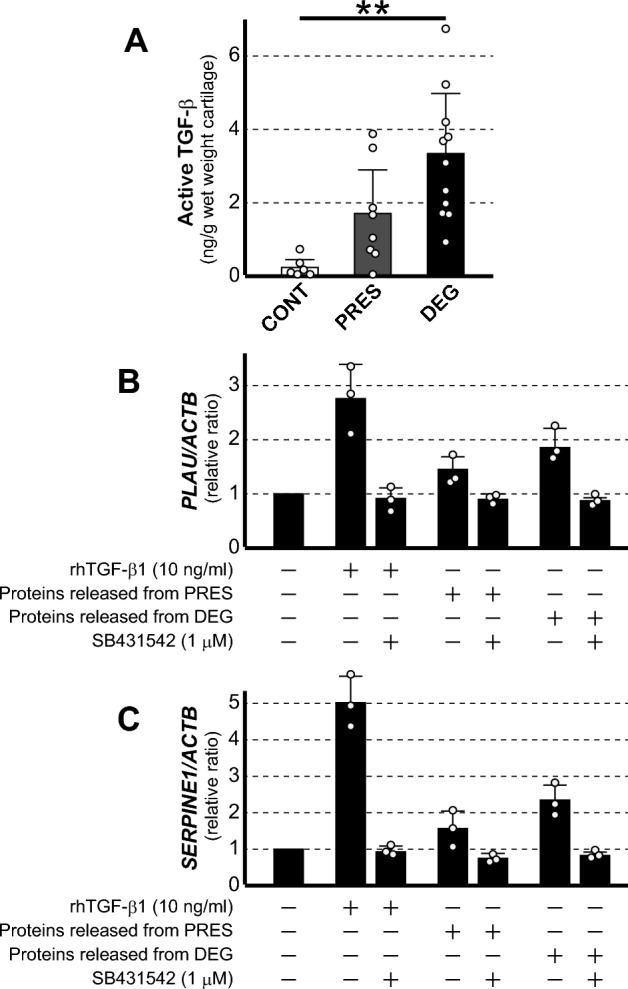
(**A**) Amounts of active TGF-β released from cartilage by loading were determined by an assay using genetically engineered HEK cells. Results of control cartilage (CONT), and OA cartilage from preserved areas (PRES) and degenerated areas (DEG) are shown by open, shaded and closed bars, respectively, which represent mean + SD of 6 or 8 cartilages. For each cartilage, assay was performed in triplicate, and their mean is shown by a dot. (**B** and **C**) Primary cultured synovial cells were cultured for 24 h in media containing either rhTGF-β1, the proteins released from PRES or those from DEG, with or without SB435142, and the expression of uPA (**B**) and PAI-1 (**C**) was evaluated. Bars represent the mean + SD of 3 independent experiments, each in triplicate, and dots indicate the mean values of respective experiments performed in triplicate. Results are shown by relative ratios against untreated cells.

In this analysis, we found that active TGF-β was released at biologically significant levels from OA cartilage. The amount released was greatest with DEG (3.50 ± 1.98 ng/g), followed by PRES (1.71 ± 1.39 ng/g), while relatively little was released from CONT (0.22 ± 0.30 ng/g) (Fig. 5A). Although there were great individual differences, the amount released from DEG was significantly greater than that from CONT. Considering the earlier result that little TGF-β2 or TGF-β3 was released from OA cartilage, the observed TGF-β activity could be entirely ascribed to TGF-β1.

We next examined whether TGF-β released from OA cartilage exhibits biological activity on synovial cells. TGF-β has been shown to induce the expression of urokinase and PAI-1 in human synovial cells^30, 31^. Based on this finding, primary cultured synovial cells were cultured in the media containing the proteins released from PRES or DEG, and urokinase and PAI-1 expression was evaluated by qPCR. In this experiment, the expression of these genes was enhanced by the addition of released proteins (Fig. 5B and C). The enhancement tended to be greater with those from DEG, which reached 1.8-fold for urokinase and 2.3-fold for PAI-1. Notably, this enhancement was entirely abrogated by a specific inhibitor of TGF-β type 1 receptor. This result indicated that TGF-β released from OA cartilage indeed exerted biological effects on human synovial cells.

## Discussion

At present, chronic, low-grade inflammation is a powerful hypothesis for OA pathology, and activation of innate immunity is a key mechanism underlying the inflammation. In particular, the activation of pattern recognition receptors (PRR) and complement system is pivotal in the innate immune response involved in chronic inflammation in OA^4, 13–15, 17^. The results of our current analyses indicated that OA cartilage releases proteins that could trigger those mechanisms.

One of the findings of this study was the release of proteins known as alarmins or damage-associated molecular patterns (DAMPs) from OA cartilage. Alarmins or DAMPs are thought to play critical roles in inflammation in OA through the activation of specific PRPs including Toll-like receptors-2 and 4^4, 13–15^. In this study, we demonstrated the release of several alarmins from OA cartilage. In OA joints, these alarmins are released by loading, primarily from DEG, and may induce inflammation in the synovium.

This study also revealed that the complement activation products C3a and C5a were released from OA cartilage by loading. The concentrations of complement proteins are known to be elevated in synovial fluid from OA knees^32, 33^, but its mechanism(s) remains to be determined. Our results indicated that diseased cartilage is a possible source of C3a and C5a in OA joints. Those proteins may be released from OA cartilage and can cause inflammation in the synovium together with alarmins.

The release of angiogenic proteins from OA cartilage was another finding of our study. Angiogenesis is a dominant feature of the synovial pathology in OA^5, 34^. In this study, we found that, under loading, at least four angiogenic proteins (FGF-1, FGF-2, VEGF-A and active TGF-β) are released in greater amounts from DEG than from CONT. Currently, angiogenesis in the synovium is considered to be induced by inflammation^5, 34^. Our current study revealed another possibility that angiogenic proteins released from diseased cartilage may also be involved in this process. It is possible that, within OA joints, these angiogenic proteins may potentiate angiogenesis induced by inflammation.

In our current study, we were surprised to find that active TGF-β was released abundantly from OA cartilage while little was released from CONT. Since the cartilage matrix contains a reservoir of latent TGF-β bound to the matrix^25^, the protein may be released upon cartilage degeneration^35^. However, whether the released TGF-β is biologically active remains unclear. To the best of our knowledge, this study is the first to demonstrate that active TGF-β is released at biologically significant levels from human OA cartilage. The biological effects of TGF-β1 on chondrocytes or synovial cells can reach a maximum at a concentration of 1–10 ng/ml^28, 30, 31^. In light of these observations, it seems plausible that active TGF-β released from OA cartilage is abundant enough to induce pathological changes in the cartilage or synovium, at least in a certain subset of OA knees.

A comparison of the result of the activity assay and the results of the quantification of acid-activated proteins by Luminex indicated that the majority of TGF-β released from DEG might be in the active form. TGF-β is known to be activated by various proteases including MMPs^29^, which are known to play dominant roles in cartilage degeneration in OA. Therefore, the result that TGF-β released from DEG was highly activated might reflect enhanced proteolysis in degenerated areas of OA cartilage.

Since TGF-β is a potent inducer of NGF in chondrocytes^28^, the above observation with TGF-β implied that NGF may be expressed within OA cartilage. However, even if NGF is expressed within OA cartilage, the protein must be released from the cartilage to generate pain, since cartilage is an aneural tissue. Thus, we examined whether NGF was released from OA cartilage, and found that NGF was released from DEG by loading. In OA joints, NGF released from OA cartilage may reach the synovium and induce pain there.

The present study was associated with several limitations. First, OA cartilage was obtained only from end-stage OA knees, and no cartilage was obtained from patients with earlier-stage disease in which the synovial changes may be more evident^36^. Second, although we applied compressive loading similar to that applied to cartilage during level walking, it is not clear how exactly our method simulated the actual in vivo situation. Third, although a proteomic analysis was performed, its value may have been limited as only two samples from each group were used for the analysis. Furthermore, while we identified the release of biologically active proteins from OA cartilage, their significance in OA pathology would be determined by their concentrations in the synovial fluid, which were not evaluated in this study. Despite these limitations, we believe that the results of this study will be of some help in dissecting the pathology of OA, a common, but highly refractory disease.

### Supplementary Information

Supplementary Information.

Supplementary Figure S1.

Supplementary Figure S2.

Supplementary Figure S3.

Supplementary Table S1.

Supplementary Table S2.

Supplementary Table S3.

Supplementary Table S4.

## Data Availability

Access to the data will be provided upon reasonable request to NF.
