# Supplementary material for: Analysis of proteins released from osteoarthritic cartilage by compressive loading
Source: Sci Rep. 2023 Oct 25;13:18292. doi: 10.1038/s41598-023-45472-x (PMC10600228; doi:10.1038/s41598-023-45472-x)
Supplement: Supplementary file 1 — Supplementary Information. [file 41598_2023_45472_MOESM1_ESM.docx]

**SUPPLEMENTARY METHODS**

**Histological evaluation**

Cartilage tissues were fixed overnight in 4% paraformaldehyde solution in PBS. The tissues were then embedded in paraffin, and 5-μm-thick sections were prepared in the direction perpendicular to the cartilage surface. The sections were stained with safranin-O/fast green and observed under a light microscope (Olympus BX51, Olympus, Tokyo, Japan).

**Quantitative proteomic analysis using iTRAQ labelling**

Approximately 180 µg of the released protein was precipitated using trichloroacetic acid (TCA) and desalted. It was then dissolved in 50 mM triethylammonium bicarbonate (TEAB) buffer, pH 8.5, containing 0.1% sodium dodecyl sulfate (SDS). Protein concentrations were determined by a bicinchoninic acid assay, and 6 µg of protein in each sample was reduced in 10 mM Tris[2-carboxyethyl]phosphine (TCEP) at 37°C for 60 mi and alkylated using 50 mM iodoacetamide for 30 min in the dark at room temperature. The proteins were then digested with sequence-modified trypsin (AB Sciex, Framingham, MA, USA). The enzyme was added to the sample at a 1:50 enzyme to protein ratio, and digestion was allowed to proceed overnight at 37°C. For each sample, the digested peptides were lyophilized, resuspended in TEBA, and labelled using an iTRAQ Reagents 4plex Applications Kit (AB Sciex) following the manufacturer’s protocol. The peptides released from the two CONT samples were labelled with iTRAQ reagents 117 and 118, respectively, whereas those from the two DEG samples were labelled with iTRAQ reagents 119 and 121, respectively.

The labelled samples were pooled, lyophilized and resuspended in 25% acetonitrile, 10 mM KH_2_PO_4_/H_3_PO_4_, pH 3.0, which were fractionated into 6 fractions using an ICAT strong cation exchange column (AB Sciex). These fractions were lyophilized and resuspended in 2% acetonitrile and 0.1% trifluoroacetic acid, and then desalted using a MonoSpin C18 column (GL Sciences, Tokyo, Japan). The fractions were then lyophilized and resuspended in 0.1% formic acid, which were subjected to an LC-MS/MS analysis.

For the LC-MS/MS analysis, high-performance liquid chromatography was conducted using an EASY-Spray 75 μm × 15 cm column (3 μm particle diameter, 100 Å pore size; ThermoFisher Scientific) on an EASY-nLC 1200 (ThermoFisher Scientific). MS/MS spectra were acquired using a Q Exactive Plus mass spectrometer (ThermoFisher Scientific) equipped with a nano-electrospray ion source, which was operated in the positive ion mode. The normalized collision energy value was set to 30%.

Survey full-scan MS spectra were obtained with a scan range of 350-1500 *m/z* and a resolution of 70,000 at *m/z* 200. The automatic gain control (AGC) target was set to 1e6 with a maximum ion injection time of 60 ms. The MS data were collected by dynamically selecting the top 10 most expressed precursor ions in the survey scan for higher energy collision dissociation (HCD) fragmentation. The HCD resolution spectra were 17,500 at *m/z* 200, the AGC target value was 5e4, and MS was dynamically excluded for 60 s.

Relative peptide quantification and protein identification were performed using Proteome Discoverer (version 2.1, ThermoFischer Scientific) with the Mascot search engine (version 2.4, Matrix Science, London, UK). Search parameters were set as follows: Species, *Homo Sapiens*; Protein database, UniProtKB/Swiss-Prot; Quantification Method, iTRAQ 4plex; enzyme, trypsin, allowing up to two missed cleavage sites; fixed modification, iTRAQ 4plex (K); variable modifications, oxidation (M) and iTRAQ4plex (Y); imputation for missing values, disabled; precursor ion mass tolerance, 10 ppm, fragment ion tolerance, 0.02 Da. Proteins were identified under the conditions of a false discovery rate (FDR) < 1% at both protein and peptide level, and matching ≥ 1 unique peptides. Considering the explanatory nature of the analysis, we did not exclude single-hit proteins from our results.

**Determination of TGF-β activity**

For this assay, released proteins were obtained directly into the culture media for HEK-Blue TGF-β cells, in place of PBS. That is, the cartilage tissues were placed and loaded in the corresponding amounts of DMEM containing 1% ITS Supplement (Sigma-Aldrich, St. Louis, MO, USA) and 1 × Penicillin-Streptomycin Solution (Sigma-Aldrich) (DMEM+ITS).

The HEK-Blue TGF-β cells were plated onto 96-well plates at a density of 5 × 10^4^ cells/well, and incubated in DMEM containing 10% fetal bovine serum (FBS, Sigma-Aldrich) and 1 × Penicillin-Streptomycin Solution overnight at 37̊°C under a humidified atmosphere with 5% CO_2_. The following day, the media were replaced with DMEM+ITS, and 24 h later, the media were replaced with DMEM+ITS containing the released proteins, or those containing graded concentrations of rhTGF-β1. The cells were cultured in these media for 24 h. Then the media were recovered, and 20 μl of each medium was mixed with 180 μl of QUANTI-Blue Solution (InvivoGen). After 30 min of incubation at 37°C, the colorimetric change in the substrate by SEAP released from the HEK-Blue TGF-β cells was quantified at 640 nm using an optical absorbance reader (iMark, BioRad, Hercules, CA, USA). The concentration of active TGF-β in the released proteins was calculated from the standard curve drawn from the absorbance of media containing graded concentrations of rhTGF-β1.

**Experiment using primary cultured synovial cells**

Synovial tissues (3-5 g) were obtained from the suprapatellar pouches of the knees with end-stage OA knees during prosthetic surgery. The tissues were finely minced with scissors, and subjected to enzymic digestion in 30 ml of DMEM containing 1% FBS, 1 mg/ml of collagenase (Sigma-Aldrich), 1 mg/ml of hyaluronidase (Sigma-Aldrich), and 200 U/ml of deoxyribonuclease I (Sigma-Aldrich) for 90 min at 37°C with gentle agitation. The cells were then washed three times with Hank’s balanced salt solution, passed through a cell strainer, and resuspended in DMEM containing 10% FBS, 0.3% L-glutamine, 1 × Penicillin-Streptomycin Solution. The cells were plated into each well of a 24-well plastic culture plate at a density of 1 × 10^6^ cells/well and cultured at 37°C in a humidified atmosphere with 5% CO_2_.

On the next day, the medium was replaced with DMEM+ITS supplemented with 0.3% L-glutamine. Twenty-four hours later, the media were replaced with fresh media containing either rhTGF-β1 (10 ng/ml), released proteins from PRES or those from DEG, with or without SB431542 (1 μM). The cells were cultured in the media for 24 h, RNA was obtained from the cells using the PureLink® RNA Mini Kit (ThermoFischer Scientific), and cDNA was generated using PrimeScript® RT Master Mix (Takara Bio, Shiga, Japan), in accordance with the manufacturers’ instructions. The expression of urokinase (*PLAU*) or PAI-1 (*SERPINE1*) was determined by qPCR using FastStart® Essential DNA Green Master (Roche Diagnostics, Basel, Switzerland) on a LightCycler® (Roche Diagnostics), with the expression of β-actin (*ACTB*) as an internal standard.
