# Supplementary material for: Analysis of proteins released from osteoarthritic cartilage by compressive loading
Source: Sci Rep. 2023 Oct 25;13:18292. doi: 10.1038/s41598-023-45472-x (PMC10600228; doi:10.1038/s41598-023-45472-x)
Supplement: Supplementary file 2 — Supplementary Figure S1. [file 41598_2023_45472_MOESM2_ESM.pdf]

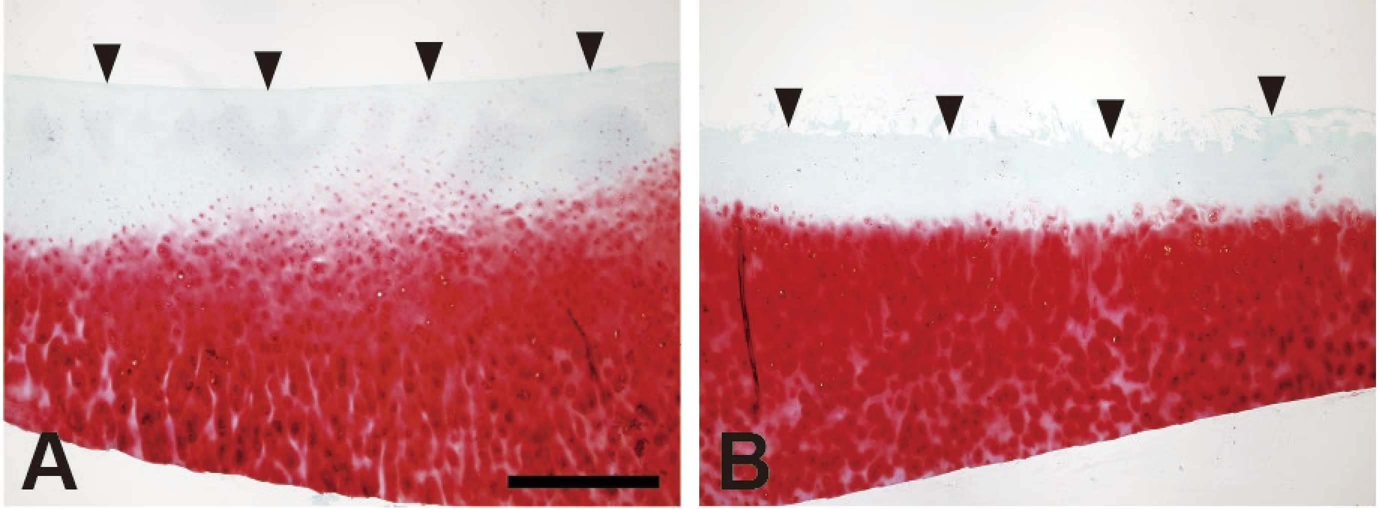

Supplementary Fig. S1. Histology of cartilage tissues that underwent compressive loading. Cartilage samples from OA knee joints were examined histologically after loading in order to validate the degree of cartilage degeneration. Representative images of the sections prepared from PRES (**A**) and DEG (**B**) are shown. Safranin O/fast green staining. Scale bar, 1 mm. Arrowheads indicate cartilage surface.
