# Supplementary material for: Analysis of proteins released from osteoarthritic cartilage by compressive loading
Source: Sci Rep. 2023 Oct 25;13:18292. doi: 10.1038/s41598-023-45472-x (PMC10600228; doi:10.1038/s41598-023-45472-x)
Supplement: Supplementary file 3 — Supplementary Figure S2. [file 41598_2023_45472_MOESM3_ESM.pdf]

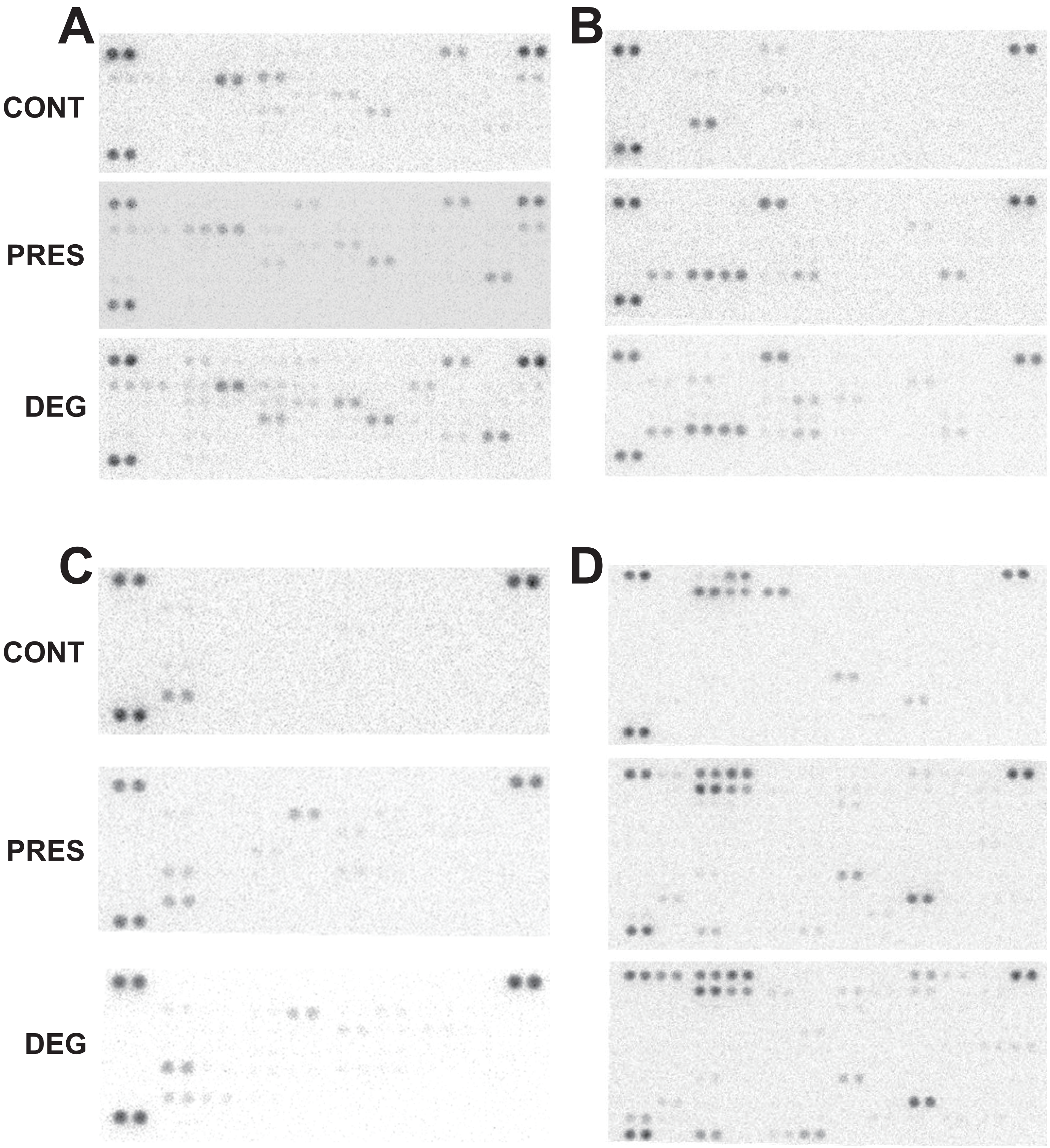

Supplementary Fig. S2. The results of the antibody array analysis. Representative results of the arrays focused on adipokines and related proteins (**A**), angiogenesis-related proteins (**B**), chemokines (**C**) and cytokines and related proteins (**D**) are shown. CONT, PRES and DEG are the results of cartilage samples obtained from control knees, and preserved areas and degenerated areas of OA knees, respectively.
