# Supplementary material for: Analysis of proteins released from osteoarthritic cartilage by compressive loading
Source: Sci Rep. 2023 Oct 25;13:18292. doi: 10.1038/s41598-023-45472-x (PMC10600228; doi:10.1038/s41598-023-45472-x)
Supplement: Supplementary file 4 — Supplementary Figure S3. [file 41598_2023_45472_MOESM4_ESM.pdf]

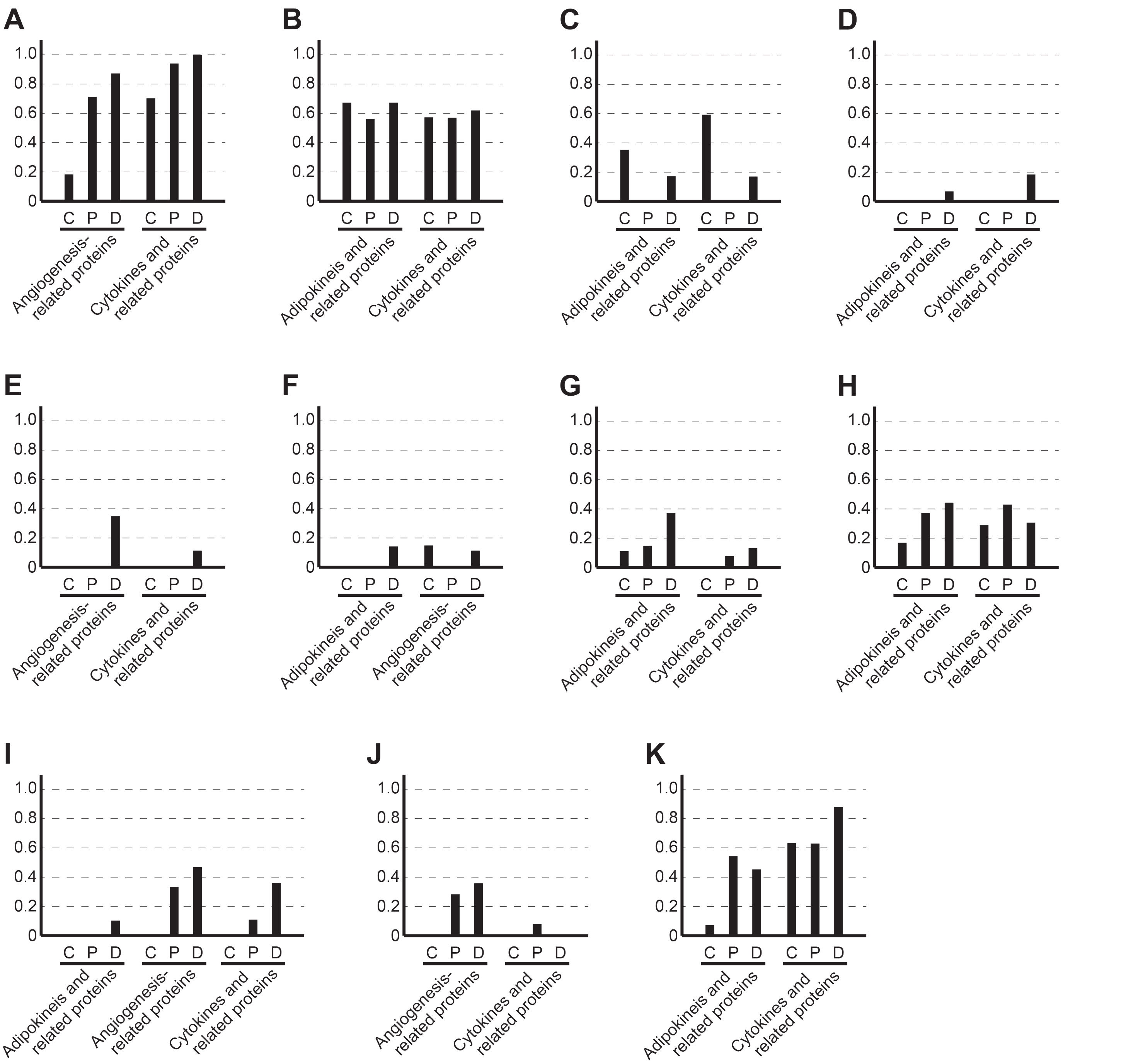

Supplementary Fig. S3. Among the 4 types of antibody arrays used in this study, antibody spots for 10 proteins were contained in 2 types of arrays, and the spot for SERPINE1 was contained in 3 types. Thus, for those 11 proteins, the results of densitometric measurements were compared across the arrays to evaluate the reproducibility of the analysis. The results of angiogenin (**A**), complement protein D (**B**), C-reactive protein (**C**), DPP4 (**D**), IGFBP-2 (**E**), IGFBP-3 (**F**), Lipocalin-2 (**G**), MIF (H), SERPINE1 (**I**), Thrombospondin-1 (**J**) and TIMP-1 (**K**) are shown. Note that those analyses were performed using respective cartilage samples from respective patients, and no cartilage samples were used for two or more antibody arrays. C, P, D indicate the results of cartilage samples obtained from control knees, and preserved and degenerated areas of OA knees, respectively. The types of antibody arrays are indicated below.
